# Perforation causing abdominal compartment syndrome after colonoscopic polypectomy: A case report

**DOI:** 10.1016/j.ijscr.2019.06.050

**Published:** 2019-07-04

**Authors:** Ying-Chun Lin, Jen-Yin Chang, Chen-Han Wu, Jian-Syun Chen, Chien-Chuan Chen

**Affiliations:** a Department of Anesthesiology, Mackay Memorial Hospital, No. 92, Sec. 2, Zhongshan N. Rd., Taipei City 10449, Taiwan; b Division of Colon and Rectal Surgery, Department of Surgery, Mackay Memorial Hospital, No. 92, Sec. 2, Zhongshan N. Rd., Taipei City 10449, Taiwan; c Mackay Medicine, Nursing and Management College, No. 92, Shengjing Rd., Beitou Dist., Taipei City 11260, Taiwan; d Mackay Medical College, No. 46, Sec. 3, Zhongzheng Rd., Sanzhi Dist., New Taipei City 25245, Taiwan

**Keywords:** Colonoscopy, Intra-abdominal hypertension, Abdominal compartment syndrome, Polypectomy, Case report

## Abstract

**Introduction:**

Bowel perforation is a potential complication of colonoscopy, and colonoscopic polypectomy has a higher risk. Perforation may be responsible for abdominal compartment syndrome (ACS), which causes multiple organ dysfunction.

Case presentation: Here, we have described the case of a woman who underwent colonoscopic polypectomy. Subsequent to the procedure, she developed progressive abdominal distension, dyspnea, drowsy consciousness, and hypotension. Her physical examination revealed increased abdominal circumference, coldness, and paleness of the lower limbs. ACS were accordingly diagnosed, most likely to have resulted from bowel perforation. Thus, decompression with a large-bore intravenous catheter and emergent laparotomy were performed. A 0.5-cm lesion over the cecum was discovered and repaired. The patient tolerated the procedure well and underwent intensive care. She was discharged on day 7 after operation, and she had completely recovered.

**Discussion:**

ACS is a rare complication followed by perforation after colonoscopy. Multiple organ dysfunction may occur from ACS, affecting the cardiovascular, pulmonary, and gastro-intestinal system. In our case, we used a small-sized catheter for decompression but in vain due to the kinking of the catheter. ACS was eventually resolved via surgical intervention. Thus, when ACS occurred, emergent decompression with an appropriate size of catheter and surgical intervention are recommended.

**Conclusion:**

Colonoscopy perforation must be considered as a contributing factor to ACS; accordingly, early recognition and intervention may avoid deterioration of ACS.

## Highlights

• Colonoscopic perforation with Abdominal compartment syndrome (ACS) is a rare complication that should be kept in mind.
• ACS deteriorates hemodynamic status and respiratory system rapidly, and may be fatal if left untreated.
• Decompression for ACS including paracentesis and surgical intervention.

## Graphical abstract

**Figure:**
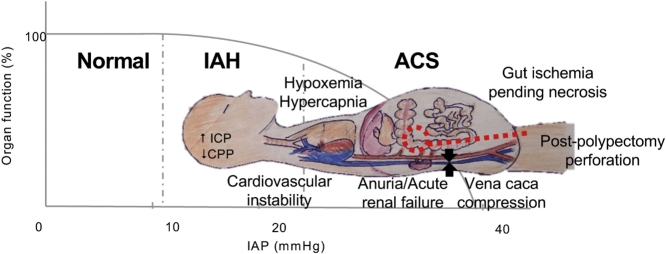

## 1 Introduction

Colorectal cancer is one of the leading causes of cancer related deaths, and colonoscopy is the standard screening and diagnostic procedure. The risk of bowel perforation varies from 0.03% to 0.13%, based on whether intervention has been performed. Patients who receive polypectomy had an incidence rate of 0.08% perforation as compared with 0.04% for those without polypectomy [1]. According to the World Society on Abdominal Compartment Syndrome (WSACS), intra-abdominal hypertension (IAH) is defined as a sustained intra-abdominal pressure (IAP) >12 mmHg, while abdominal compartment syndrome (ACS) is defined as IAP > 20 mmHg with end-organ dysfunction [2]. Most patients were critically ill and unable to convey symptoms. The physical signs observed were tensely distended abdomen, oliguria, hypotension, dyspnea, elevated jugular venous pressure, or acute pulmonary decompensation. Definitive diagnosis of ACS requires the measurement of the IAP, which is measured by using intragastric, intracolonic, intravesical (bladder), or inferior vena cava catheters [3]. Surgical decompression is the definitive management of IAH and ACS; therefore, further supportive care is considered [2]. The work has been reported in line with the SCARE 2018 statement [4].

## 2 Presentation of case

**Fig. 1:**
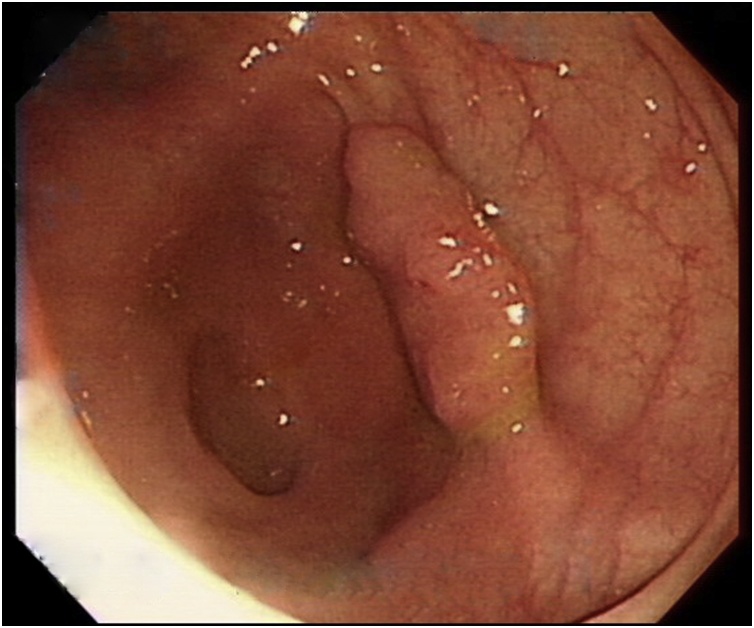
**Colonoscopy.** One 1.5 cm sessile type polyp was discovered over cecum.

**Fig. 2:**
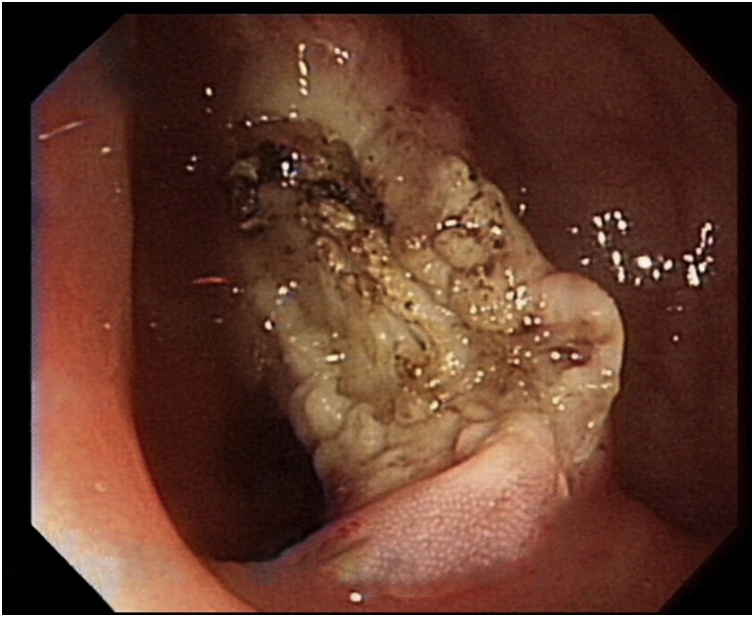
**After polypectomy.** Polypectomy was performed and bowel wall damage was noted.

A 47-year-old woman without any medical diseases underwent colonoscopy due to positive occult blood test during health surveillance. She had undergone laparoscopic appendectomy due to appendicitis previously. Her recent complete blood count, biochemistry, coagulation profile, electrocardiogram, and chest X-ray were normal. Prior to the colonoscopy, her vital signs were heart rate (HR) 72 beats per minute (bpm), blood pressure (BP) 139/74 mmHg, and oxygen saturation (SpO_2_) 100% in room air. Under intravenous general anesthesia, colonoscopy revealed a 1.5-cm cecal polyp at the cecum (Fig. 1). We accordingly performed the polypectomy (Fig. 2); however, bowel wall damage occurred, and perforation was suspected. Since there was no accompanying situation to help confirm the decision, we ended the procedure and intended to obtain informed consent after the patient awaked. Vital signs of the patient were stable during the entire process.

While arriving at the post-anesthetic care unit (PACU), she had not yet regained her consciousness; however, her vital signs were relatively stable (BP 95/56 mmHg, HR 70 bpm, RR 12/min, SpO_2_ 100%). After 10 min, her consciousness remained drowsy, with no response to stimuli and then gradually distended, tense abdomen was noted with dropped BP as 80/56 mmHg. She also showed rapid and shallow breathing, and mottled and cyanosis of the extremities and lips. Her physical signs revealed tympanic percussion and increased abdominal circumstances. Assisted ventilation was supported at that time. Under the suspicion of ACS, urgent needle decompression with a 20-gauge intravenous catheter was introduced at the right lower quadrant of her abdomen. An immediate rush out of air indicated the presence of IAH. However, kinking of the catheter resulted in incomplete drainage of free air, and the distended abdomen persisted. Concerning the impaired cardiovascular and respiratory functions, we performed emergent laparotomy and colon repair under general anesthesia with endotracheal tube intubation. The ventilation was extremely difficult, with diminished bilateral breath sounds and steady pulse oximeter reading. The initial peak airway pressure was approximately 30 cmH_2_O with a low tidal volume (approximately 200 mL) and improved dramatically while the pressure was relieved by laparotomy. The peak airway pressure became approximately 18 cmH_2_O and the tidal volume improved to approximately 450 mL. We repaired the cecal perforation, and the patient tolerated the entire process well. She was discharged on day 7 of the surgery with complete recovery. There was no functional impairment discovered at the outpatient clinic after 6 months of discharge.

## 3 Discussion

The risk of bowel perforation during colonoscopy varies from 0.03% to 0.13%, and patients receiving polypectomy have an higher incidence of perforation (0.08%). [1]. Factors related to perforation, include patient-related risk factors, (such as old age, male gender, comorbidities, inflammatory colonic disease, use of drugs like warfarin, NSAIDs, and steroids [5]), procedural-related factors, and lesion-related factors, (such as morphology, right-sided lesion [6], submucosal fibrosis [7], sessile polyps, and lesions sized >2 cm) [5,8,9].

Colonoscopic perforation can be managed by conservative treatment, endoscopic clipping, and surgical intervention, including primary closure, bowel resection, and fecal diversion [10,11]. A previous case report has also revealed successful treatment with polyglycolic acid sheets and fibrin glue [12]. Currently, the numbers of patients undergoing surgical treatment remain greater than those undergoing endoscopic treatment [10]. Notably, the conversion from endoscopic treatment to surgical treatment, older age (>60 years), and high-grade American Society of Anesthesiologists classification (≥3) have relevant to poor outcomes [10].

ACS is a rare complication that may complicate colonoscopic perforation. A few case reports documented this complication with varied severity ranging from hypertensive pneumoperitoneum to cardiac arrest [[13], [14], [15]]. ACS affects various organ systems. Increased IAP may impair respiratory function by decreasing the functional residual capacity and tidal volume with increased airway pressure due to the cephalad pressure on the diaphragm. IAH and ACS impair the cardiac output with hypotension by interfering with the venous return and right heart filling due to compression of the inferior vena cava and the elevated intra-thoracic pressure. In addition, ACS impairs perfusion of the intra-abdominal organs, including the kidney, hepatobiliary system, and intestine, resulting in oliguria, activation of renin–angiotensin–aldosterone system, metabolic acidosis, bowel edema and intraluminal bacteria translocation, and worsening ACS [16].

To normalize the IAP, IAH could be treated with diuresis, paracentesis, and/or decompressive laparotomy based on the severity [16]. The WSACS guideline suggests paracentesis of obvious intra-abdominal fluid due to readily accessible technique comparing open laparotomy [3]. Nevertheless, acute ACS caused by colonoscopic perforation is not discussed in the guideline. All 3 case reports noted dramatic improvement in the respiratory function and hemodynamic status after single or double paracentesis during CPR. Two of the patients received surgery after stabilization, and one of them was discharged after 10 days of observation without further intervention [[13], [14], [15]].

However, our patient failed to recover from paracentesis and, subsequently, received emergent laparotomy. The major difference is between the size of catheter used in our case (20 gauge) and that used in all previous case reports mentioned (14 gauge) for paracentesis. The inadequate size of catheter is insufficient for decompression and is prone to interruption due to kinking. Another difference is our patient suffered from progressive increase of IAP and deterioration of vital signs after cessation of the examination, while others reported sudden changes during the procedure. Since the effect of the insufflation type in sedated patients was conflicting [17,18], we used air rather than carbon dioxide (CO_2_) during the procedure. Compared to CO_2_, air is absorbed slowly through the bowel mucosa, causing more luminal distension and potentially greater abdominal pain. This fact may explain why the patient had a delayed onset of ACS at PACU.

Thus, early recognition and intervention are essential whenever ACS occurs.

## 4 Conclusion

Colonoscopic perforation with ACS is a rare complication that should be kept in mind. ACS deteriorates rapidly; therefore, early diagnosis and management are crucial.

## Conflicts of interest

There is no conflicts of interest.

## Author contribution

Ying-Chun Lin: Writing – Original Draft

Jen-Yin Chang: Writing – Review & Editing, investigation

Chen-Han Wu: Data Curation, Supervision, Investigation

Jian-Syun Chen: Investigation, Writing – Review & Editing

Chien-Chuan Chen: Supervision

## Consent

Written informed consent was obtained from the patient. Upon request, copy of the written consent is available for review by the Editor-in-Chief of this journal.

## Ethical approval

Our study is exempted from ethical approval in Institutional Review Board of our hospital.

## Provenance and peer review

Not commissioned, externally peer-reviewed.

## Registration of research studies

This is a case report and not a human study. It is exempt from registering.

## Guarantor

Jen-Yin Chang

Ying-Chun Lin

Chien-Chuan Chen
